# Physiological lentiviral vectors for the generation of improved CAR-T cells

**DOI:** 10.1016/j.omto.2022.05.003

**Published:** 2022-05-18

**Authors:** María Tristán-Manzano, Noelia Maldonado-Pérez, Pedro Justicia-Lirio, Pilar Muñoz, Marina Cortijo-Gutiérrez, Kristina Pavlovic, Rosario Jiménez-Moreno, Sonia Nogueras, M. Dolores Carmona, Sabina Sánchez-Hernández, Araceli Aguilar-González, María Castella, Manel Juan, Concepción Marañón, Juan Antonio Marchal, Karim Benabdellah, Concha Herrera, Francisco Martin

**Affiliations:** 1 Department of Genomic Medicine, Pfizer-University of Granada-Andalusian Regional Government Centre for Genomics and Oncological Research (GENYO), PTS, Avda. de la Ilustración 114, 18016 Granada, Spain; 2 LentiStem Biotech, Pfizer-University of Granada-Junta de Andalucía Centre for Genomics and Oncological Research (GENYO), PTS, Avda. de la Ilustración 114, 18016 Granada, Spain; 3 Department of Cellular Biology, Faculty of Sciences, University of Granada, Campus Fuentenueva, 18071 Granada, Spain; 4 Maimonides Institute of Biomedical Research in Córdoba (IMIBIC), Cellular Therapy Unit, Reina Sofia University Hospital, University of Córdoba, 14004 Córdoba, Spain; 5 Department of Medicinal and Organic Chemistry, Faculty of Pharmacy, University of Granada, Campus Cartuja, 18071 Granada, Spain; 6 Department of Hematology, ICMHO, Hospital Clínic de Barcelona, Villarroel 170, 08036 Barcelona, Spain; 7 Biosanitary Research Institute of Granada (ibs.GRANADA), Department of Human Anatomy and Embryology, Faculty of Medicine, University of Granada, Granada 18071, Spain; 8 Departamento de Bioquimica y Biología Molecular 3 e Inmunología, Facultad de Medicina, Universidad de Granada, Avda. de la Investigacion 11, 18071 Granada, Spain

**Keywords:** CAR-T, physiological expression, TCR-like expression, lentiviral vectors, leukemia, lymphoma, exhaustion, WAS gene promoter, tonic signaling, T cells phenotype

## Abstract

Anti-CD19 chimeric antigen receptor (CAR)-T cells have achieved impressive outcomes for the treatment of relapsed and refractory B-lineage neoplasms. However, important limitations still remain due to severe adverse events (i.e., cytokine release syndrome and neuroinflammation) and relapse of 40%–50% of the treated patients. Most CAR-T cells are generated using retroviral vectors with strong promoters that lead to high CAR expression levels, tonic signaling, premature exhaustion, and overstimulation, reducing efficacy and increasing side effects. Here, we show that lentiviral vectors (LVs) expressing the transgene through a *WAS* gene promoter (AW-LVs) closely mimic the T cell receptor (TCR)/CD3 expression kinetic upon stimulation. These AW-LVs can generate improved CAR-T cells as a consequence of their moderate and TCR-like expression profile. Compared with CAR-T cells generated with human elongation factor α (EF1α)-driven-LVs, AW-CAR-T cells exhibited lower tonic signaling, higher proportion of naive and stem cell memory T cells, less exhausted phenotype, and milder secretion of tumor necrosis factor alpha (TNF-α) and interferon (IFN)-ɣ after efficient destruction of CD19^+^ lymphoma cells, both *in vitro* and *in vivo*. Moreover, we also showed their improved efficiency using an *in vitro* CD19^+^ pancreatic tumor model. We finally demonstrated the feasibility of large-scale manufacturing of AW-CAR-T cells in guanosine monophosphate (GMP)-like conditions. Based on these data, we propose the use of AW-LVs for the generation of improved CAR-T products.

## Graphical abstract

**Figure:**
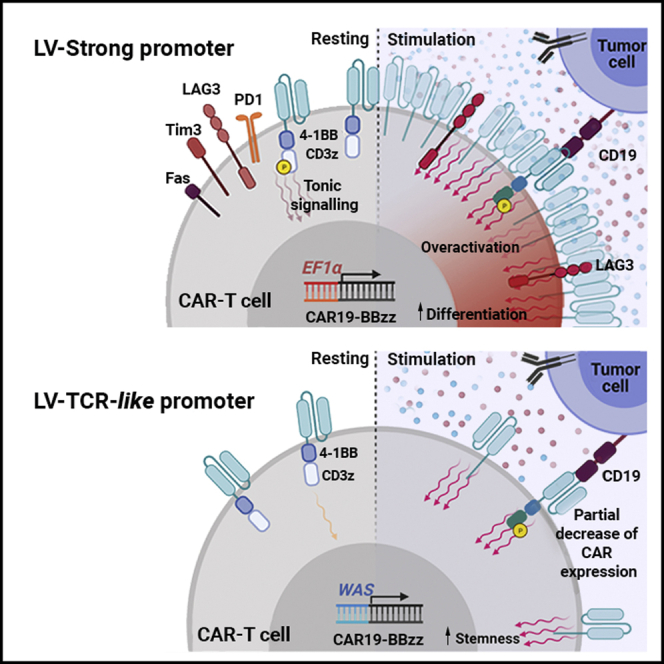

## Teaser

Tristán-Manzano and colleagues showed that physiological AWARI CAR-T cells can be efficiently produced at large scale and present lower tonic signaling, a more stem phenotype, and moderate secretion of pro-inflammatory cytokines while maintaining or improving their anti-tumoral activity compared with state-of-the-art ARI CAR-T cells.

## Introduction

Chimeric antigen receptor (CAR) T cells have become one of the most promising approaches for the treatment of cancer in particular for B-lineage neoplasms, as emphasized by the approval of four αCD19-CAR-T medicaments by the US Food and Drug Administration (FDA) and/or the European Medicines Agency (EMA): tisagenlecleucel (Kymriah, Novartis), axicabtagene ciloleucel (Yescarta, Kite-Gilead), brexucabtagene autoleucel (Tecartus, Kite-Gilead), and lisocabtagene maraleucel (Breyanzi, Bristol Myers Squibb). However, in spite of the impressive results of αCD19-CAR-T cells, there are still several aspects that must be improved. Aggressive severe side effects due to CAR-T overstimulation, such as cytokine release syndrome (CRS) and neuroinflammation, are common among treated patients and can lead to fatal outcomes. Furthermore, sustained complete remissions range from 62% to 42% for patients treated with commercial advanced therapy medicinal products (ATMPs), evidencing much room for improvement.^1^

Different works have uncovered the importance of controlling CAR expression levels at the surface of the CAR-T cells in order to optimize its therapeutic activity.^2^, ^3^, ^4^ In spite of this, all four approved αCD19 ATMPs and most of those that are been tested in ongoing clinical trials use autologous T cells transduced with retroviral vectors expressing an αCD19-CAR through constitutive, strong promoters, such as the human elongation factor α (EF1α) and the murine stem cell virus (MSCV) long terminal repeat (LTR). High CARs concentrations on the T cell surface can result in spontaneous clustering of the CARs (independent of the ligand), leading to tonic signaling.^4^^,^^5^ This tonic signaling can affect safety (pro-inflammatory cytokines secretion in non-target tissues) and efficacy (due to premature exhaustion or Fas/FasL-induced cell death) of CAR-T cells.^3^, ^4^, ^5^ Furthermore, high-density and constitutive CAR presence on the T cell membrane can also lead to overstimulation upon antigen recognition that also affects safety (excess of pro-inflammatory cytokine secretion) and efficacy (early exhaustion, apoptosis, and loss of T naive and stem cell memory [T_N/SCM_] phenotype) of the CAR-T cells.^2^^,^^3^ In this direction, Eyquem et al.^2^ generated CAR-T cells expressing the αCD19-28ζ CAR through the endogenous T cell receptor constant alpha chain (TRAC) locus using CRISPR-Cas9. In this manuscript, the authors concluded that tight transcriptional regulation of CAR expression, lowering CAR levels upon target binding and recovering 2 to 3 days later, was critical for optimal CAR-T performance. However, despite the great potential of genome-editing tools for therapeutic applications, there are still several technological and safety issues that need to be solved before the approval of genome-edited cells as medicaments.

Contrary to genome-editing tools, lentiviral vectors (LVs) derived from HIV-1 have already been approved by the FDA and EMA (Kymriah, Breyanzi, and Zynteglo). Latest generation LVs are very resistant to transgene silencing^6^^,^^7^ and allow the control of the transgene through physiological or drug-inducible promoters.^8^, ^9^, ^10^, ^11^, ^12^, ^13^, ^14^, ^15^, ^16^ We therefore searched for LVs that express transgenes on T cells following the expression kinetic of the T cell receptor (TCR) after stimulation. Since the TRAC promoter in human mature T cells is not well characterized, we focus on a well-defined promoter that controls the expression of the WAS protein (WASP), which is involved in the formation of the immunological synapse and in translating TCR signals to several T cell functions.^17^^,^^18^ We reason that *WAS*-promoter-driven LVs^19^, ^20^, ^21^ could be an interesting option to express CARs due to their moderate expression levels and the functional relationship with the TCR. Here, we showed that, indeed, *WAS*-promoter-driven LVs partially mimicked the TCR expression kinetic both in EGFP- and CAR-expressing LVs, with a small down-regulation upon stimulation and recovering basal levels (prior stimulation) in 5–7 days. Based on these data, we generated TCR-like CAR-T cells using LVs and analyzed potential improvements compared with standard CAR-T cells expressing the CAR through strong promoters. TCR-like expression of a αCD19-CAR led to lower tonic signaling in the absence of antigen as well as a better response in the presence of lymphoma cells, with lower exhaustion markers, lower pro-inflammatory cytokine secretion, and a more stem phenotype both *in vitro* and *in vivo*. However, the *in vitro* and *in vivo* anti-lymphoma activities were comparable between EF1α-driven and TCR-like driven CAR-T cells, probably due to the limitations of the model. Interestingly, increased anti-tumor efficacy could be observed when TCR-like driven CAR-T cells were used in an artificial CD19^+^ pancreatic tumor model.

## Results

### *WAS*-promoter-driven LVs mimic the TCR expression pattern upon T cell activation

**Figure 1:**
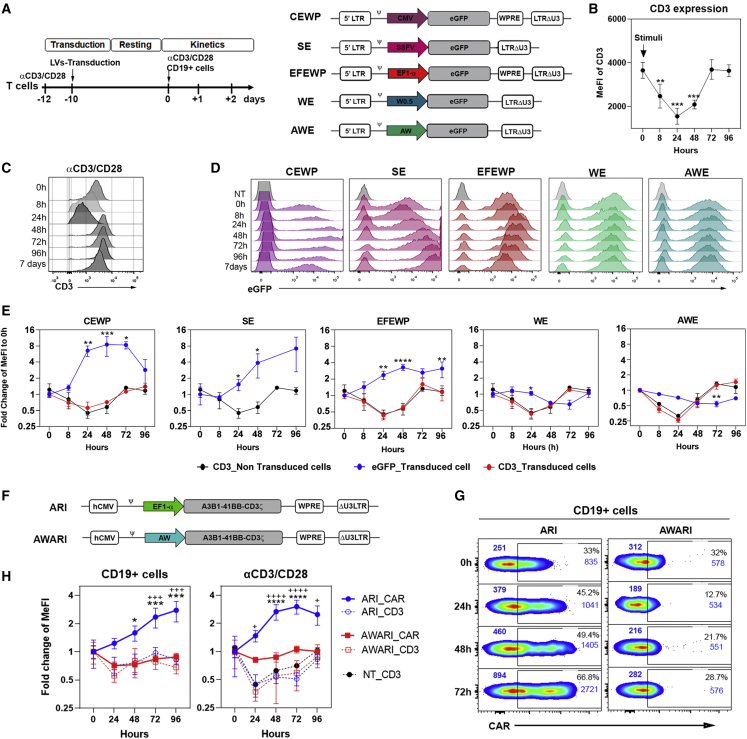
T cells transduced with *WAS* promoter-driven LVs mimic the TCR expression pattern (A) (Left) Timeline of the experiments to study EGFP expression pattern after TCR and CAR stimulation. (Right) Diagrams of the different EGFP-expressing LVs are shown. CMV, SFFV, EF1α, W0.5, and AW promoters were used in CEWP, SE, EFEWP, WE, and AWE LVs, respectively (see Materials and methods for details). (B) CD3 MeFI of T cells stimulated with αCD3/CD28 and measured at the indicated time points is shown. ∗p < 0.05; one-way-ANOVA; n ≥ 5. (C) Representative histograms of CD3 expression on T cells at different times post-stimulation are shown. (D) Representative histograms show EGFP expression levels on T cells transduced with strong-promoter-driven (CEWP, SE, and EFEWP) and *WAS*-promoter-driven (WE and AWE) LVs at different times post-stimulation with αCD3/CD28. (E) Graphs show fold change in EGFP expression (blue line) and CD3 expression on non-transduced (black lines) and transduced (red lines) T cells along time after αCD3/CD28 stimulation. Two-way ANOVA, multiple comparison post-test, compared with CD3 expression pattern of non-transduced T cells (NT_CD3): ∗p < 0.05; ∗∗p < 0.01; ∗∗∗p < 0.001; ∗∗∗∗p< 0.0001 . (F) Diagrams of the EF1α-promoter-driven (ARI) and *WAS*-promoter-driven (AWARI) LVs expressing the ARI-0001 CAR (αCD19 A3B1-41BB-CD3ζ) are shown. (G) Representative dot plots of CAR expression levels on CAR-T cells generated with the ARI (left) or AWARI (right) LVs measured at different time points after activation with CD19^+^ cells are shown. MeFIs of total population and CAR^+^ (gate) populations are indicated in blue at top left or top right of each plot, respectively. (H) Fold change of CAR (solid lines) and CD3 (dashed lines) expression of ARI CAR-T (blue lines) and AWARI CAR-T (red lines) cells at different time points after activation with CD19^+^ cells (left) or αCD3/CD28 nanomatrix (right) is shown. Data show the MeFI of bulk population at different time points relative to those at 0 h. Populations with similar percentage of CAR^+^ cells (mean ± SEM; ARI versus AWARI, 33.5% ± 0.9% versus 30.1% ± 1.2%; p *=* 0.12) and vcn (ARI: 2.7 ± 1 c/c and AWARI: 5.9 ± 2.7 c/c; p *=* 0.34) were compared. Two-way ANOVA; ∗p < 0.05; ∗∗∗p < 0.001; ∗∗∗∗p < 0.0001 for ARI_CAR compared with AWARI_CAR and +p < 0.05, +^++^p < 0.001; ++++p < 0.0001 for comparison of ARI_CAR to ARI_CD3, AWARI_CD3, or NT_CD3 (n ≥ 5).

As LVs have already been approved by EMA and the FDA agencies, LVs expressing the CARs with a similar kinetic to the TCR (TCR-like LVs) could be a real alternative to existing LVs to generate more potent and safer CAR-T cells. We first investigated whether *WAS*-promoter-driven LVs could have a more physiological expression pattern compared with current LVs used for CAR-T cells generation. We generated EGFP-expressing LVs particles from two different *WAS*-promoter-driven LVs (Figure 1A, right, WE and AWE)^19^^,^^20^ and three LVs driven by strong promoters (Figure 1A, right): CEWP, SE, and EFEWP, expressing EGFP through the cytomegalovirus (CMV), spleen focus forming virus (SFFV) promoter, and the EF1α promoters, respectively.

In order to analyze whether the *WAS*-promoter-driven LVs mimic the TCR expression pattern, we first studied the CD3 expression levels of primary human T cells after stimulation with αCD3/CD28 nanomatrix at different time points (Figures 1A [left], 1B, 1C, and S1A). In parallel, T cells transduced with the different EGFP-expressing LVs were stimulated and analyzed by flow cytometry at the same time points (Figure 1D). As it has been reported previously,^2^ CD3 expression in T cells decreased soon after stimulation, reaching minimal expression at 24 h and 48 h at both protein and mRNA levels and recovering basal levels (prior stimulation) after 5–7 days (Figures 1B, 1C, and S1A). Contrary to the CD3 expression pattern, all LVs harboring constitutive-strong promoters, such as EF1α (EFEWP), SFFV (SE), and CMV (CEWP), increased EGFP expression after T cell stimulation (Figures 1D [pink, purple, and red histograms] and 1E [blue lines of CEWP, SE, and EFEWP graphs]). Interestingly, *WAS*-promoter-driven LVs partially mimicked the TCR expression kinetic, repressing EGFP expression upon stimulation and recovering basal levels after 4–7 days (Figures 1D [green and blue histograms] and 1E [blue lines of WE and AWE graphs]). Importantly, CD3 expression pattern was not affected by LVs transduction (Figure 1E, compare black lines with red lines). Since EGFP is an intracellular and stable protein, we did not expect a strong down-regulation in this time frame. Moreover, the reduction in CD3 that is observed at initial stages of activation is due to internalization of CD3, something that cannot be observed using EGFP.

Since similar results were obtained with both WE and AWE-LVs, AW promoter (which includes the W0.5 promoter from WE together with 0.38 kb from the alternative promoter of the WAS locus) was selected. We next investigated whether CARs expressed by *WAS*-promoter-driven LVs also followed a TCR-like kinetic. We constructed the AWARI-LV (see Materials and methods for details) based on the ARI-0001 LV backbone (Figure 1F),^22^ a CAR19-BBzz that uses the A1B6 clone as scFv and has been recently approved as an advanced therapy drug in Spain for the treatment of patients over 25 years of age with acute lymphoblastic leukemia. We then generated ARI and AWARI CAR-T cells expressing similar CAR levels and analyzed their CAR and CD3 expression kinetic upon activation with CD19^+^ cells (Figures 1G, 1H [left], S1B [left], S1C, and S1D [left]) or αCD3/CD28 (Figures 1H [right], S1B [right], and S1D [right]). Interestingly, AWARI CAR-T cells showed a down-regulation of the CAR levels after antigenic stimulation and a recovery after 48–72 h, mimicking quite closely the CD3 behavior (Figures 1G [right plots] and 1H [red-solid squares]), while ARI CAR-T cells showed a clear increment on the CAR levels after T cell activation (Figures 1G [left plots] and 1H [blue-solid circles]), which significantly differed from the CD3 profile after TCR and CAR axis stimulation of the same cells (Figure 1H, dash lines, red-empty circles, and blue-empty squares). Of note, CD3 expression pattern in unmodified T cells was only affected by αCD3/CD28 stimulation, while CAR-expressing T cells responded similarly to both αCD3/CD28 and CD19 stimulation (Figure S1D). Altogether, our results showed that the AW-LVs can be used to generate CAR-T cells expressing the CAR in a TCR-like manner.

### WAS-driven expression of CAR improves characteristics of CAR-T cell products

**Figure 2:**
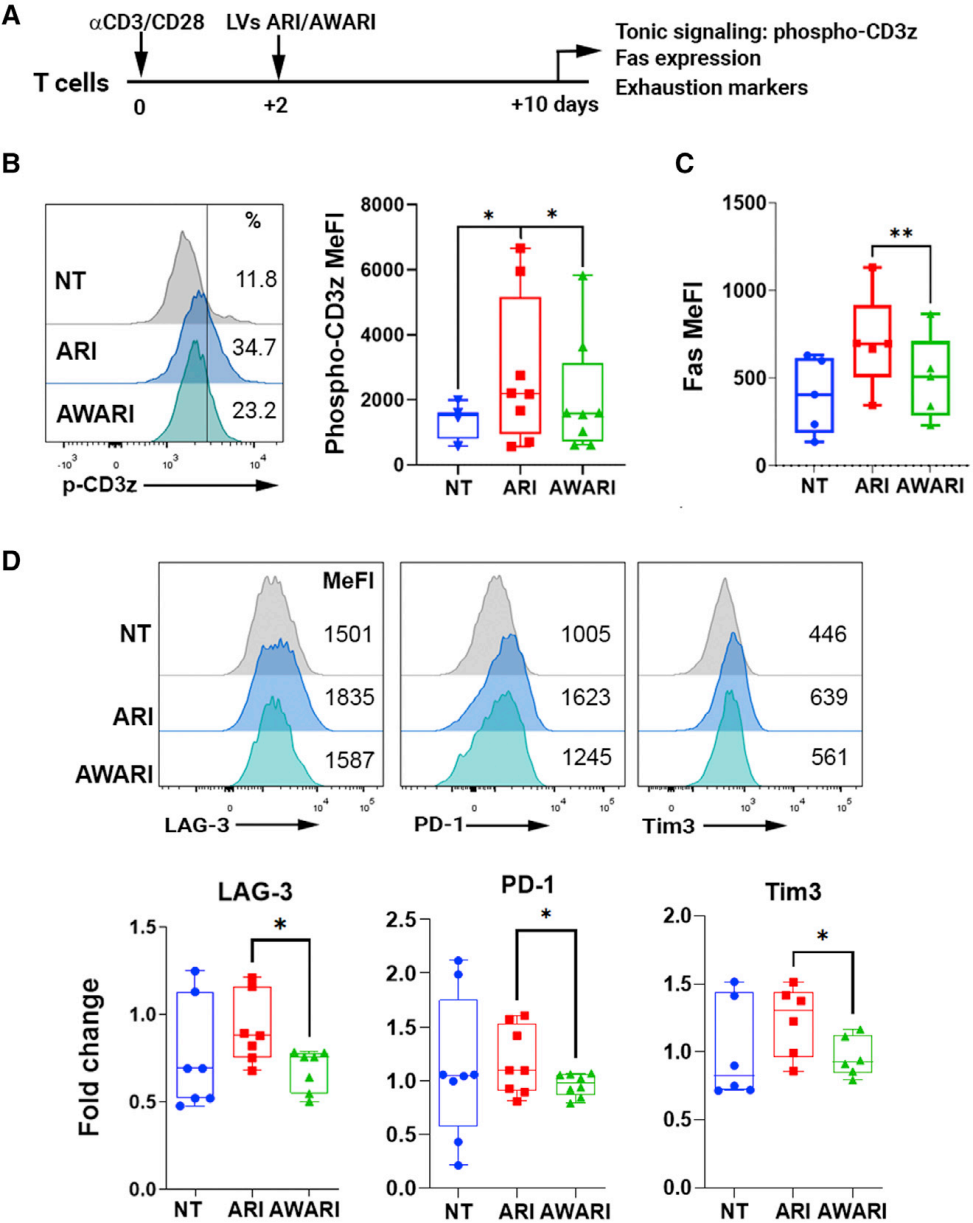
AWARI CAR-T cells maintain fitness at resting state (A) Diagram of the procedure used to investigate CAR-T cell products fitness. The analyses were performed on T cells transduced at similar efficiency. %CAR+: ARI = 27.0% ± 3.8% and AWARI = 23.4% ± 3.7%; VCN: ARI = 3.1 ± 1.1 vcn/c and AWARI = 2.9 ± 1.1. (B) Left: representative histograms show phospho-CD3z (p-CD3z) expression of NT (gray), ARI (blue), and AWARI (green) CAR-T cells. Right: graphical representation of pCD3z MeFI levels of NT (blue), ARI (red), and AWARI (green) CAR-T cells is shown. Two-tailed paired t test is shown (four independent donors; n ≥ 5). ∗p < 0.05. (C) Graph shows Fas (CD95) MeFI levels of NT (blue), ARI (red), and AWARI (green) CAR-T cells. Two-tailed paired t test (n = 4 and 3 independent donors). ∗∗p < 0.01. (D) Top: representative histograms of LAG-3, PD1, and Tim3 expression of NT (gray), ARI (blue), and AWARI (green) CAR-T cells are shown. Bottom: fold change of LAG-3, PD-1, and Tim3 expression levels of ARI (red) and AWARI (green) CAR-T cells related to the expression in NT cells (blue) is shown. Data are MeFI ± SEM. Two-tailed paired t test is shown (four independent donors; n ≥ 5), ∗p < 0.05.

In addition to antigen-driven stimulation, CARs can also produce antigen-independent tonic signaling in CAR-T cells that depend on the CAR configuration, surface density, and the self-aggregating properties of each CAR.^3^^,^^4^^,^^23^ Enhanced tonic signaling can result in antigen-independent expansion of T cells, leading to their differentiation and exhaustion. In addition, tonic signaling of 4-1BB CARs results in the induction of Fas and FasL that make T cells susceptible to activation-induced cell death (AICD). Since the AW promoter, in addition to achieving a TCR-like expression pattern, also expresses lower CARs levels (see Figure 1G), we expect lower tonic signaling and exhaustion markers of AWARI versus ARI CAR-T cells when analyzed in a CD19-independent context. To verify this hypothesis, ARI and AWARI CAR-T cells (harboring equivalent vcn/c) were left to rest at basal state (day 10 after initial αCD3/CD28 activation) (Figure 2A) and the expression of phospho-CD3z (as a marker of tonic signaling) (Figure 2B), Fas (as an indicator of AICD) (Figure 2C), and LAG-3, PD-1, and Tim3 (as exhaustion markers) (Figure 2D) were analyzed. As expected, AWARI CAR-T cells expressed significant lower levels of pCD3z, Fas, LAG-3, PD-1, and Tim3, which suggests that TCR-like and/or moderate levels of CAR expression at basal state can preserve several desirable characteristics of the CAR-T product in the absence of antigenic stimulation.

### AWARI CAR-T cells maintain a more stem phenotype and a moderate secretion of pro-inflammatory cytokines after efficient killing of CD19^+^ cells

**Figure 3:**
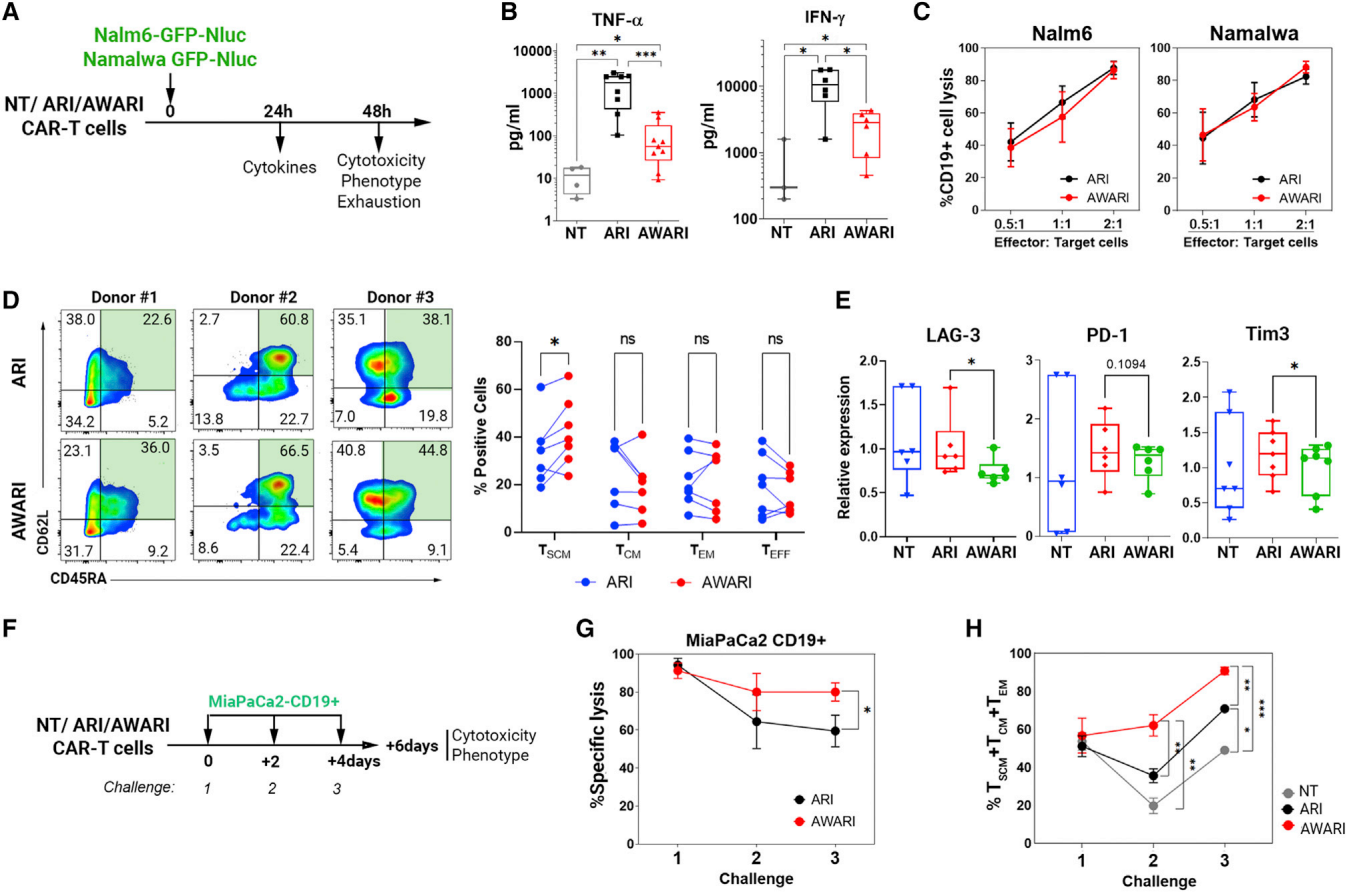
AWARI CAR-T cells maintain improved properties after elimination of CD19^+^ cells *in vitro* (A) Work flow of the *in vitro* cytotoxic evaluation of NT, ARI, and AWARI CAR-T cells. The analyses were performed using T cells transduced at similar efficiency (%CAR^+^: ARI = 27.8% ± 3.9% and AWARI = 23.6% ± 2.7%; VCN: ARI = 2.1 ± 0.8 c/c and AWARI = 5.4 ± 1c/c). (B) Levels of secreted TNF-α (left) and IFN-ɣ (right) produced by NT, ARI, and AWARI CAR-T cells after 24 h of stimulation with Namalwa at ratio 1:1 are shown (four independent donors, n ≥ 4 for TNF-α and three independent donors, n ≥ 3 for IFN-ɣ). Mann-Whitney t test is shown. ∗p < 0.05; ∗∗p < 0.01; ∗∗∗p < 0.001. (C) Lysis of CD19^+^ cells (Nalm6 and Namalwa) at different ratios of co-culture with ARI and AWARI CAR-T cells during 48 h is shown. Percentage of lysis is related to NT-driven, non-specific CD19^+^ lysis (n = 5). Non-significance in two-way ANOVA test is shown. (D) (Left) Representative CD45RA/CD62L dot plots of different ARI and AWARI CAR-T cells generated from three different donors after 48 h of co-culture with Namalwa cells at ratio 1:1 are shown. (Right) Graphical representation of the different subpopulations (T_SCM_ in green) in bulk AWARI CAR-T cells compared with ARI CAR-T cells after 48 h in the presence of Namalwa cells is shown. Two-tailed paired T test. ns, not significant. (E) Relative expression levels of LAG-3, PD-1, and Tim3 of ARI (red) and AWARI (green) CAR-T cells relative to NT (blue) 48 h after co-culture with Namalwa cells are shown. Data represent MeFI of each marker in ARI or AWARI CAR-T cells divided by the MeFI in NT cells. One-tailed paired t test is shown (n ≥ 5). (F) Diagram of the experiment performed with the CD19^+^ pancreatic cellular model is shown (MiaPaCa2 CD19^+^). NT, ARI, and AWARI CAR-T cells were challenged with MiaPaCa2 CD19^+^ three times at an initial ratio of 5:1 (CAR-T:target cells) every 2 days. (G) Specific lysis of MiaPaca2 CD19^+^ with ARI and AWARI CAR-T cells at each challenge is shown (two independent donors; n = 4 one-tailed paired t test). (H) Percentage of T_SCM_ + T_CM_ + T_EM_ of ARI (black) and AWARI (red) after the different challenges with MiaPaCa2 is shown. Multiple Tukey’s comparison is shown.

The loss of efficacy of CAR-T comes in part from the early exhaustion of the CAR-T cells due to an excess of CAR signaling after antigen encounter.^24^ Overstimulation of CAR-T cells can also generate CRS, promoted by increased pro-inflammatory cytokines release. We therefore evaluated the cytotoxic potential, tumor necrosis factor alpha (TNF-α) and interferon (IFN)-ɣ secretion, phenotype, and expression of exhaustion markers of ARI and AWARI CAR-T cells. Two tumoral CD19^+^ models, Nalm6-GFP-Nluc (B cell acute lymphoblastic leukemia [B-ALL] derived) and Namalwa-GFP-Nluc (Burkitt’s lymphoma derived) were incubated with both CAR-T cells and the different parameters analyzed as indicated in Figure 3A. Interestingly, AWARI CAR-T cells secreted lower levels of TNF-α and IFN-γ compared with ARI cells (Figure 3B) but maintain a similar anti-tumoral efficacy against Nalm6 and Namalwa (Figure 3C). We also observed that AWARI CAR-T cells retained a less differentiated phenotype (T_SCM_, CD45RA^+^CD62L^+^) of the different donors compared with ARI CAR-T cells after killing Namalwa cells (Figure 3D). Interestingly, LAG-3 and Tim3 expression levels were significantly lower in AWARI compared with ARI CAR-T cells, particularly after killing Namalwa cells (Figure 3E), but no differences were observed for PD-1 or Tim3 against the Nalm6 cell model (Figure S2A). In summary, here, we show that, in spite of the similar anti-lymphoma activity, AWARI CAR-T cells exhibit a milder secretion of TNF-α and IFN-ɣ as well as reduction in LAG-3 expression, both important players in CRS.

### TCR-like expression of the CAR improves anti-tumor efficacy in CD19^+^ pancreatic tumor cellular model

In spite of a more stem phenotype of the TCR-like CAR-T cells (AWARI) compared with EF1α-driven CAR-T cells (ARI), the anti-lymphoma activity was comparable between both CAR-T cells, even after different encounters with Namalwa cells (Figure S2B). In order to analyze whether the improvement in stemness observed in TCR-like CAR-T cells could also lead to an improvement in killing efficacy, we generated a pancreatic tumor cellular line (MiaPaCa2) expressing CD19 and GFP-Nluc (Figure S3). This cellular model could represent a more complex environment in which CAR-T cells with a stem phenotype can also show a more efficient tumor killing. Therefore, MiaPaCa2-GFP-Nluc was confronted with NT, ARI, and AWARI T cells at three consecutive times to favor long-lasting CAR-T cells (Figure 3F). Killing activity and T cell phenotype were measured after each challenge. Interestingly, compared with ARI, AWARI CAR-T cells resulted in enhanced specific lysis after three consecutive challenges (Figure 3G) with an enrichment of fewer effector populations (Figure 3H).

### AWARI CAR-T cells are as efficient as ARI CAR-T cells in the eradication of CD19^+^ lymphoma cells *in vivo* and maintain a more stem-like phenotype

**Figure 4:**
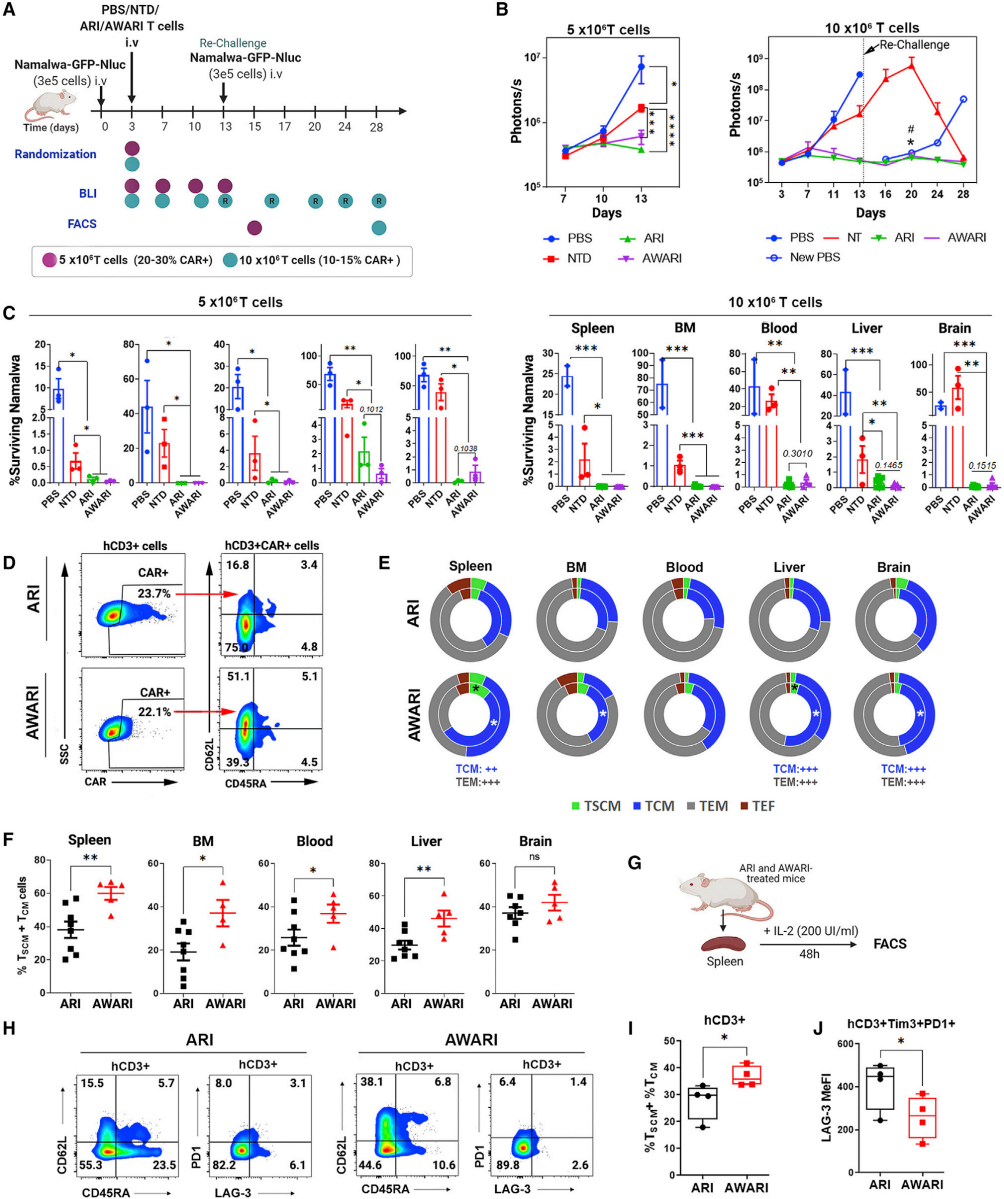
*In vivo* anti-tumor activity and phenotype of ARI and AWARI CAR-T cells (A) Experimental design of *in vivo* anti-tumor activity and phenotypic studies of 5 × 10^6^ (purple dots, 25.1% of ARI^+^ cells and 22.3% of AWARI CAR-T cells: ∼1.26 and 1.12 × 10^6^ CAR-T cells, respectively; VCN of ARI = 17.8 c/c and AWARI 13.8 c/c) and 10 × 10^6^ (blue dots, 14.7% of ARI^+^ cells and 13.0% of AWARI^+^ cells: ∼1.47 and −1.3 × 10^6^ CAR-T cells; VCN of ARI = 3.6 c/c and AWARI = 5.8c/c) of T cells transduced with ARI and AWARI-LVs. Rechallenge (a new dose of Namalwa-GFP-Nluc was inoculated) is indicated with an arrow at day 13 in the 10 × 10^6^ dose experiment. Flow cytometry (FACS) analysis was performed after sacrifice at day 13 for the 5 × 10^6^ experiment and at day 28 for 10 × 10^6^ experiment. (B) Tumor bioluminescence (BLI) progression with 5 × 10^6^ (left) and 10 × 10^6^ (right) T cells doses at different days post-Namalwa inoculation is shown. “New PBS” indicated new untreated NSG mice inoculated with Namalwa as controls of the rechallenge. Two-way ANOVA-Tukey’s multiple comparisons are shown. ∗p < 0.05; ∗∗∗p < 0.001; ∗∗∗∗p < 0.001. In the 10 × 10^6^ graph, ∗p < 0.001 NT vs ARI and #p <0.001 NT vs AWARI. (C) Percentage of surviving Namalwa-GFP-Nluc cells in bone marrow (BM), spleen, blood, liver, and blood determined by FACS after the sacrifice with the low (left panels) and high (right panels) doses is shown. This value is analyzed in the “singlets human cells” gate (see Materials and methods). One-tailed Mann-Whitney t test; ∗p < 0.05, ∗∗p < 0.01, and ∗∗∗p < 0.001. N (mice) of low T dose: PBS = 3, NT = 3, ARI = 3, and AWARI = 3; and high T dose: PBS = 2, NT = 3, ARI = 8, and AWARI = 5. (D) Representative dot plots of CAR expression and phenotype of CAR^+^ cells of gated hCD3 from the spleen of ARI and AWARI mice after sacrifice of the high-dose experiment are shown. (E) Graphical representation of proportions of T_N_/T_SCM_ (CD62L^+^CD45RA^+^) (green), T_CM_ (CD62L^+^CD45RA^−^) (blue), T_EM_ (CD62L^−^CD45RA^−^) (gray), and T_EF_ (CD62L^−^CD45RA^+^) (red) subpopulations in ARI (top) and AWARI (bottom) CAR^+^ cells in the indicated organs after sacrifice of challenged (outer circle) and rechallenged (inner circle) mice is shown. ∗p < 0.05 when comparing the T cells subsets of rechallenged ARI and AWARI treated mice. Mann-Whitney one-tail is shown. ++p < 0.01 and +++p < 0.001 of two-way ANOVA, Bonferroni post-test of all the population of the total mice ARI = 8 (3+5R); AWARI = 5 (2+3R). (F) Percentage of T_N_/T_SCM_ and T_CM_ after sacrifice of ARI and AWARI hCD3^+^CAR^+^ cells is shown (ARI = 8; AWARI = 5; one-tailed T test). (G) Spleens from treated mice were isolated, and cell suspensions were cultured with 200 IU/mL of IL-2 in TexMACS medium and 2% P/S during 48 h. (H) Representative dot plots of CD45RA/CD62L and PD1/LAG3 populations from spleens of ARI and AWARI mice are shown. (I) Percentage of T_N_/T_SCM_ and T_CM_ populations in the CD3^+^ population after 48 h in culture is shown. Two-tailed t test is shown (n = 4). (J) LAG-3 MeFI of the CD3^+^Tim3^+^PD-1^+^ cells is shown. One-tailed t test is shown (n = 4).

We next performed a direct comparison of the anti-tumor activity of ARI versus AWARI CAR-T cells in a xenograft mice model of human Burkitt’s lymphoma using two different T cells doses and a rechallenge after first lymphoma cells clearance (Figure 4A; see Materials and methods for details). Both CAR-T cells efficiently eliminated Namalwa tumor cells in treated mice with both doses (Figures 4B, 4C, and S4A), and all survived free of the disease (Figure S4B). Of note, tumor clearance was observed in some mice inoculated with NT cells in the 10 × 10^6^ T cells, probably due to a strong xenograft response as indicated also by strong graft versus host disease (GVHD) symptoms (data not shown).

However, a small amount of Namalwa cells (<5%) were detected in the liver of some ARI mice and in the brain of some AWARI mice treated particularly with the low dose (Figure 4C, left panels). Importantly, CAR expression driven by the two vectors was maintained in all the organs after sacrifice (Figures 4D and S4C), even with a second challenge, when compared with those *ex vivo* levels prior to infusion (Figure S4C), indicating no promoter silencing.

After showing the same therapeutic efficacy, we investigated potential differences in CAR-T cell phenotype after tumor clearance. Interestingly, AWARI CAR-T cells showed a higher proportion of memory (T_CM:_ CD45RA^−^CD62L^+^) and stem memory (T_SCM_: CD45RA^+^CD62L^+^) (Figure 4D) cells in spleen, bone marrow (BM), blood, and liver when compared with ARI-treated mice (Figures 4E and 4F) as well as increased expression of CD62L (Figures S4C and S4D). Especially after rechallenge (inner circle, Figure 4E), T_SCM_ population of AWARI CAR-T cells was increased in spleen and liver and T_CM_ subset in BM and brain. In order to further characterize the potential differences, we cultured the cells from processed spleens during 48 h *in vitro* with 200 IU/mL of interleukin-2 (IL-2) in TexMACs medium (Figure 4G), and we confirmed an increment in T cell memory populations in AWARI-derived spleens (Figures 4H and 4I) and the lower expression of exhaustion markers (PD-1^+^TIM3^+^LAG3^+^) (Figures 4H and 4J).

### Large-scale semi-automated manufacturing of AWARI CAR-T cells

**Figure 5:**
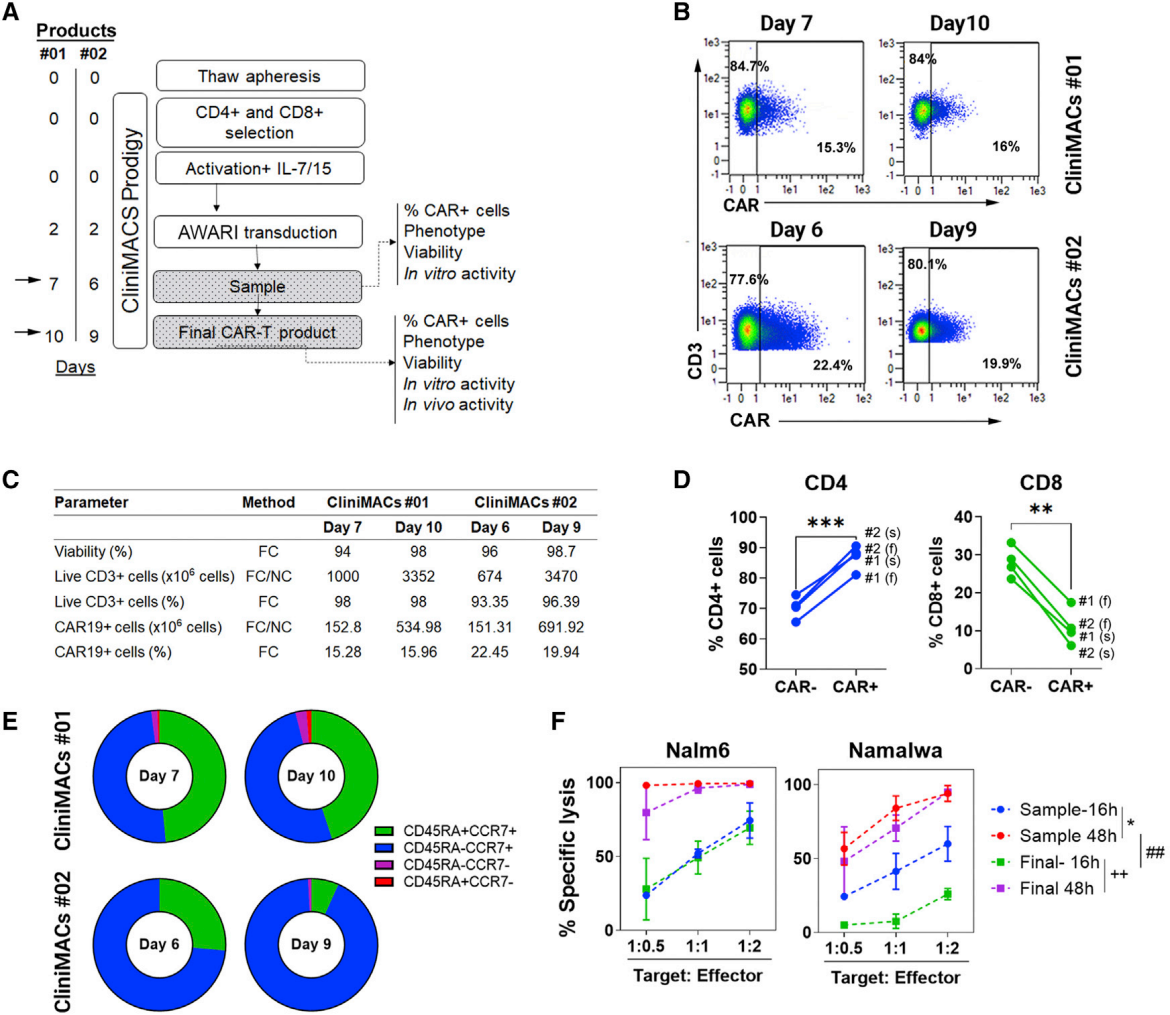
GMP-like large-scale production of AWARI CAR T cells (A) Timeline of the GMP-like batches no. 01 and no. 02 of AWARI CAR-T cells in the CliniMACS Prodigy. (B) Dot plots of CAR expression at days of sample (7 and 6) and final product (10 and 9) for both no. 01 (left) and no. 02 (right) batches are shown. (C) Data from viability, number of CD3, efficiency of CAR-T cell transduction, and number of obtained CAR-T cells are shown. FC, flow cytometry; NC, Neubauer chamber counting. (D) Variation of percentage of CD4 and CD8^+^ cells in CAR^−^ and CAR^+^ cells at sample (s) and final products (f) is shown. Two-tailed t test is shown. ∗∗p < 0.001; ∗∗∗p < 0.001. (E) Graphical representation of the T cells populations at sample (days 6 or 7) and final products (day 9 or 10) is shown. T_N_/T_SCM_, naive and stem cell memory (CD45RA^+^CCR7^+^); T_CM_, central memory (CD45RA^−^CCR7^+^); T_EM_, effector memory (CD45RA^−^CCR7^−^); and T_EMRA_, effector memory RA^+^ (CD45RA^+^CCR7^−^). (F) *In vitro* specific lysis of CD19^+^ cells Nalm6 (left) and Namalwa (right) CD19^+^ cells by AWARI CAR-T cells harvested at sample day (red and blue lines) and final day (green and violet lines) for productions no. 01 and no. 02 is shown. CAR-T cells products were co-cultured at different ratios during 16 h (blue or green lines) or 48 h (red or violet lines). Two-way ANOVA-Tukey’s multiple comparisons are shown.∗p < 0.05; ++p < 0.01; ##p < 0.01.

Before translation of AW-LV to ATMP production for clinical applications, it is important to demonstrate their performance for large-scale automated manufacturing. We therefore used laboratory grade AWARI-LVs to generate two batches of CAR-T cells (no. 01 and no. 02) in the CliniMACs Prodigy Bioreactor (Miltenyi Biotec) under good manufacturing practice (GMP) conditions (see Materials and methods). Samples at days 7 and 10 for batch no. 01 and days 6 and 9 for batch no. 02 (Figure 5A) were collected to study CAR expression (Figure 5B), viability, expansion (Figure 5C), CD4/CD8 ratio (Figure 5D), phenotype (Figure 5E), and *in vitro* lytic activity (Figure 5F). In both productions processes, cells were efficiently expanded, with more CD4^+^ than CD8^+^ CAR^+^ cells and prominent T_N_/_SCM_ (CCR7^+^CD62L^+^) subset (44.85% ± 0.07%) at day 7 or day 6, which were drastically reduced at final day of the production (16.55% ± 13.93%), turning into T_CM_ cells but with minimal effector and effector memory populations (Figure 5E). As described for other CAR-T cell products, lower expansion times rendered more potent AWARI CAR-T cells (Figure 5F). All these results point to the AWARI-LVs as a valuable tool for a future clinical trial, although improvements in its lentiviral backbone could further increase transduction efficacy in order to lower costs and time of production.

### Large-scale manufactured AWARI CAR-T cells products are highly efficient for tumor clearance and maintain good levels of T_N/SCM_ cells *in vivo*

**Figure 6:**
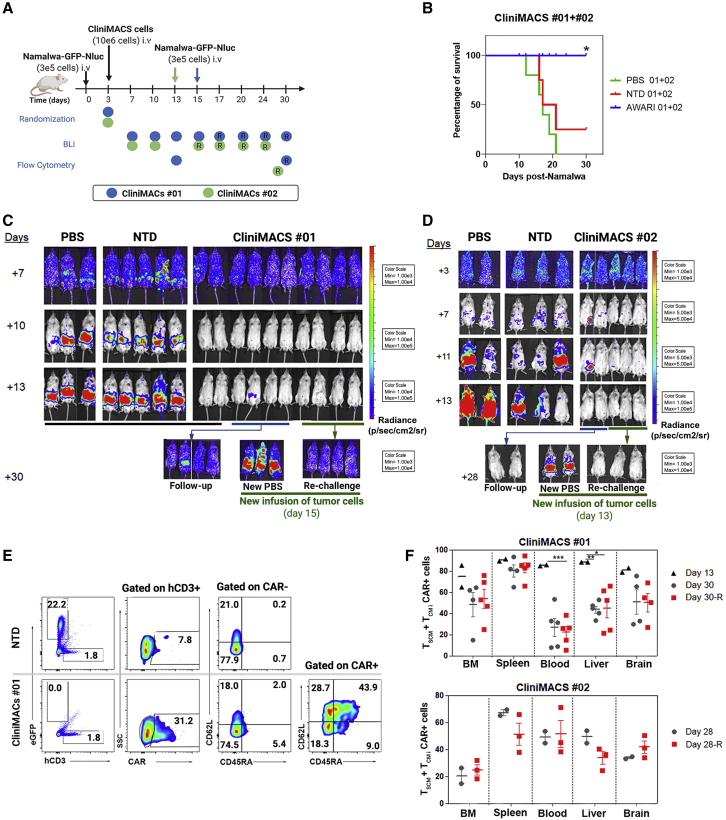
*In vivo* anti-tumor activity and phenotype of CliniMACS-produced AWARI CAR-T cells after one or two Namalwa challenges (A) Experimental design and timeline of the two experiments with CliniMACS batches no. 01 and no. 02. R indicates rechallenge. (B) Survival of PBS, NT, or AWARI-inoculated mice of no. 01 and no. 02 products is shown. Log rank Mantel-Cox test is shown. ∗p < 0.05 (C and D) Representative BLI images of tumor burden measured in no. 01 (C) and no. 02 (D) experiments at different days post-Namalwa inoculation as indicated are shown. In both studies, at day 15 (C) or 13 (D), new Namalwa cells were inoculated to AWARI-treated mice (rechallenge, green line) and to new control mice (“new PBS”). The rest of the treated mice (four AWARI mice in C and two AWARI mice in D) were kept for a follow-up (blue line, follow-up group). (E) Representative dot plots of Namalwa/hCD3 presence, CAR^+^ cells of hCD3, and phenotype of CAR^−^ and CAR^+^ of NT cells and AWARI CliniMACS no. 01 from BM after sacrifice are shown. (F) Percentage of T_N_/_SCM_ + T_CM_ population in hCD3^+^CAR^+^ cells analyzed in different organs from mice with one or two (rechallenge, R) inoculations of Namalwa tumor cells in no. 01 (left) and no. 02 (right) CliniMACS batches on the final day is shown. Only when CAR^+^ population was >1%, data were analyzed and included here. N (CliniMACS no. 01): PBS = 3, NT = 5, and AWARI = 10 (two sacrificed on day 13; four mice with one- and four mice with two challenges). N (CliniMACS no. 02): PBS = 2, NT = 3, and AWARI = 5 (2 + 3 rechallenged mice). Two-way ANOVA and Bonferroni post-test are shown; ∗p < 0.05, ∗∗p < 0.001, and ∗∗∗p < 0.0001.

Finally, we performed a detailed analysis of large-scale manufactured AWARI CAR-T cells in terms of their *in vivo* anti-tumor efficacy and their phenotype after isolation from treated mice (Figure 6) for both no. 01 (blue dots) and no. 02 (green dots) CliniMACS batches. Anti-tumor activity of both AWARI CAR-T cells products were analyzed in the Namalwa xenograft mice model as depicted in Figure 6A. Survival (Figure 6B) and tumor progression (Figures 6C and 6D) were monitored up to 28–30 days post-treatment, showing a strong anti-tumor activity of the AWARI CAR-T cells with 100% survival and undetectable tumor cells in treated mice (Figures 6C, 6D, and S5A). Control PBS and non-transduced (NTD) inoculated mice developed the disease at similar rates, and the majority required to be sacrificed for compassionate reasons at days 13–16. At this time point, the stemness of human CAR-T cells of two AWARI-treated mice (Figure 6F, top panel) and CD62L expression (Figure S5C) in no. 01 experiment was clearly maintained. New Namalwa cells were inoculated in AWARI CAR-T-cells-treated mice and also in new mice (“new PBS”). Rechallenged AWARI mice remained tumor free through the duration of the experiment, as did the follow-up group. Similar results were observed in both productions (Figures 6C, 6D, and S5A). The percentage of CAR^+^ cells (Figure 6E) was maintained before (*ex vivo*) and after *in vivo* elimination of Namalwa cells in the xenograft model with a slight increase in some of the organs (Figure S5B). Importantly, the phenotypic analysis confirmed the presence of a high proportion of T_SCM_/T_CM_ populations (Figures 6E, 6F, and S5D) at the endpoint of rechallenged and non-rechallenged mice. However, a significant reduction of these populations was observed from day 13 to day 30 (Figures 6F, top panel, and S4C). These data confirmed the *in vitro* (Figure 3) and *in vivo* (Figure 4) results, showing that the expression of the ARI-0001 through a TCR-like promoter (AWARI CAR-T cells) maintains the proportion of T_SCM_/T_CM_ CAR-T cells upon antigen encounter.

## Discussion

The therapeutic benefits in R/R B lineage lymphoid neoplasms had led to the approval of different CAR-T products and over 800 clinical trials around the globe by mainly using autologous T cells modified with retrovirus-based vectors to express the CAR through a strong promoter (such as EF1α or MSCV promoters). Extrinsic parameters (tumor burden and target density) and intrinsic characteristics of the CAR structure (scFV, hinge/spacer, costimulatory domains, bispecific or multi-specific CARs, etc.), T cell composition, and the type of vector clearly influence the clinical outcome of the CAR-T cell therapy. Until very recently, the highest effort in modifications has been focused on the CAR protein level in order to improve the current limitations of this therapy and often pays less attention to how this protein is expressed in the target cells in different situations.

There are several evidences indicating that high levels and continuous expression of the CAR can be deleterious for the efficacy and safety of the CAR-T products.^2^, ^3^, ^4^^,^^25^^,^^26^ The loss of efficacy comes in part from the early exhaustion and/or apoptosis of the CAR-T cells due to an excess of CAR signaling after antigen encounter.^24^ Strong and unregulated CAR expression also increase tonic signaling in the absence of target antigen,^4^ promoting exhaustion.^3^^,^^5^ On the other side, safety issues due to CAR over-expression relate to the increased antigen-independent tonic signaling and to overstimulation of CAR-T cells that generate CRS. Using genome-editing technologies, Eyquem et al.^2^ demonstrated that TCR-like expression of CARs resulted in lower tonic signaling, reduced CAR-T exhaustion, and increased proportion of T_SCM_/T_N_ CAR-T cells, resulting in improved anti-tumor activity. These authors also showed that low expression was not enough to achieve optimal CAR-T products, concluding that a down-regulation of the CAR upon antigen encounter was required for optimal CAR-T performance. This is in line with the work of Weber and colleagues,^25^ who propose that transient rest in the CAR signaling can prevent or reverse exhaustion, restoring the anti-tumor functionality and promoting a memory-like phenotype.

In this manuscript, we investigated whether LVs can be used to obtain TCR-like expression kinetics that could also result in an improved CAR-T cell product. Our search for human promoters that could mimic the TCR expression pattern pointed to the TRAC locus; however, the TRAC promoter in mature human T cells is not well defined. We therefore focus on the *WAS* gene promoter because WASP is only expressed in hematopoietic cells, is involved in the formation of the immunological synapse, and acts as an adaptor of TCR signals.^17^^,^^18^ We have previously developed different *WAS*-gene-based promoters and identified the AW promoter, harboring fragments from the alternative and proximal promoter, as the best option to express transgenes in hematopoietic cells,^19^, ^20^, ^21^ which makes it an attractive candidate for T cells expression. Here, we showed that, indeed, *WAS*-promoter-driven LVs (AW-LVs) partially mimicked the TCR (CD3)-expression kinetic, with a small down-regulation upon stimulation and recovered basal levels in 5–7 days. We also showed that, as observed in untransduced T cells, CD3 expression in CAR-T cells (either EF1-α or *WAS*-driven CAR-T cells) was also down-modulated after CAR and TCR engagement. This TCR down-modulation of CAR-T cells can occur through direct engagement of the TCR (αCD3/CD28) and/or in an independent manner, probably influenced by tyrosine kinase signaling, which is additionally provided by the CD3z domain of the CAR.^27^

Based on the ability of AW-LVs to mimic TCR expression pattern on T cells, we investigated whether 4-1BB-CAR-T cells generated with these “physiological” LVs could be a better CAR-T cell product compared with those generated with LVs harboring strong or viral promoters. We analyzed the CAR-T cells characteristics before (mimicking the CAR-T product prior the infusion) and after antigen encounter (analyzing anti-tumor efficacy and phenotype). Although, in terms of *in vitro* or *in vivo* anti-tumor activity, we could not detect significant differences with EF1α-driven CAR-T cells against the CD19^+^ lymphoma model, AW-CAR-T cells exhibited lower tonic signaling and a less differentiated phenotype after efficient killing of CD19^+^ cells, with reduced secretion of pro-inflammatory cytokines. These data are largely in agreement with the observations of Eyquem et al.,^2^ using genome-editing technologies to express the CAR through the TRAC locus. However, these authors also showed improved *in vivo* anti-tumor activity. The differences in the tumor model (Nalm6 versus Namalwa) as well as in the CAR signaling domain (CD28 versus 4-1BB) and scFv (FMC63 versus A3B1) could explain the observed differences. Indeed, the potential deleterious effects of CAR over-expression and tonic signaling are highly influenced by the CAR configuration.^2^^,^^4^ For instance, replacing CD28 with the 4-1BB costimulatory domain reversed exhaustion in CAR T cells and improved its persistence and therapeutic efficacy.^23^ The good characteristics of EF1α-driven 4-1BB-CAR-T cells and the experimental limitations derived by the GVHD caused by the administration of the CAR-T cells can explain the absence of increased therapeutic efficacy in our animal models. On the other hand, we observed increased anti-tumor potential due to the implementation of the AW promoter against an artificial CD19^+^ model of pancreatic carcinoma *in vitro*, which confirms the potential benefits of this TCR-like promoter in a different context. These data raise a new scenario, where a non-continuous expression and lower density of the CAR can be potentially beneficial in other cancer types, as Rodriguez-Marquez et al.^26^ have observed in αBCMA-CAR-T cells therapy multiple myeloma. In this work, they compared high- and low-CAR-density populations, observing that high-density CAR-T cells increase tonic signaling and effector signature and correlated with a worse clinical response.

In spite of the good behavior of 4-1BB-CARs, Gomes-Silva et al.^4^ demonstrated that very high expression levels of 4-1BB–CARs also lead to apoptosis (AICD) and limit CAR-T cells expansion. The same authors also showed that lowering expression levels by using EF1α-driven LVs instead of an LTR-driven ɣ-retroviral vector, they observed an improvement in the T cell’s stemness, expansion potential, and therapeutic efficacy. These studies showed that EF1α-driven LVs already generate a good CAR product for some 4-1BB-CARs. Here, we show that TCR-like expression can further improve 4-1BB-CAR-T products, in particular for the ARI-0001, by maintaining a stem phenotype, controlling tonic signaling and pro-inflammatory cytokine secretion, and exhibiting lower levels of Fas at basal state, which can prevent an excessive AICD.

An important aspect to consider in CAR-T cells are the potential side effects due to overstimulation and/or inadequate stimulation. Severe CRS (grades 3 or 4) is an important hallmark in CD19^+^ that can compromise efficacy and lead to life-threatening conditions.^28^^,^^29^ Upon tumor cell encounter, CAR-T cells release different pro-inflammatory cytokines, such as TNF-α, IFN-ɣ, IL-1, and granulocyte-macrophage colony-stimulating factor (GM-CSF), that leads to macrophage recruitment and activation, causing a dangerous cytokine storm involving IL-6 and GM-CSF.^30^^,^^31^ It has also been demonstrated that activation of macrophages through CD40/CD40L, CD69, and LAG-3 also contributes to massive activation.^29^ Although CRS can be managed with corticosteroids and anti-IL-6R (tocilizumab), these interventions also block T cell activation, compromising CAR-T efficacy.^29^ In this manuscript, we have shown that CAR-T cells generated with AW-LVs exhibit a milder secretion of TNF-α and IFN-ɣ after tumor cells encounter, which agrees with the low-density CAR’s results against multiple myeloma.^26^ In addition, we noted that LAG-3 expression was reduced after Namalwa and Nalm6 interaction, which suggests that AW-CAR-T cells could lower the risk of CRS through direct and indirect signaling.

We finally investigated the feasibility of generating a viable CAR-T cell product for clinical translation. Here, we have demonstrated that it is feasible to generate TCR-like CAR-T cells in a CliniMACs Prodigy, with high cell viability, predominant stem and central memory populations, and potent *in vitro* and *in vivo* anti-tumor activity. On the other hand, ARI-0001 generated more memory and effector memory population,^32^ highlighting another potential advantage of AW-CAR-T cells during the manufacturing process. In contrast, the efficacy of transduction was lower compared with ARI (∼18.4% versus ∼30.6%), suggesting that a future optimization of the AWARI-LV backbone (e.g., searching and eliminating new splice-donor and splice-acceptor sites generated in the AWARI construct) could further improve the manufacturing process of AW-CAR-T cells.

Taken all together, we propose the use of AW-LVs as an alternative platform for the manufacturing of CAR-T cells in order to provide better efficacies and/or safety of the final products. The final benefits of expressing different CARs through the AW promoter will need to be determined for each indication.

## Materials and methods

### Cell culture

Nalm6 (ATCC CRL-3273), Namalwa (ATCC CRL-1432), Jurkat (ATCC TIB-152), HL-60 (ATCC CCL-240), HEK-293T (ATCC CRL-11268), and MiaPaCa2 (ATCC CRL-1420) cells were cultured as described elsewhere. Namalwa and Nalm6 were modified to express enhanced GFP (EGFP) and Nanoluciferase (NanoLuc) using the SELWP, as described previously.^33^ In addition, MiaPaCa2 cells were first transduced with SELWP and then with EhCD19-LVs to express EGFP-NanoLuc and human CD19.

### Human samples

Primary T cells were isolated from fresh or frozen apheresis products obtained from healthy donors at the Hematology Department of the Hospital Universitario Reina Sofía (Córdoba, Spain). All donors gave their written informed consent, and the study was performed according to the guidelines of the local ethics committee and complies with the requirements regarding quality and safety for donation, obtaining, storage, distribution, and preservation of human cells and tissues under the Spanish-specific regulation (RD-L 9/2014). Pan-T cells were isolated by negative selection using immunomagnetic beads (Pan T cell Isolation Kit, Miltenyi Biotec, Bergisch Gladbach, Germany) and following MACSExpress Separator (Miltenyi Biotec) or AutoMACs Pro Separator’s (Miltenyi Biotec) protocol and cultured in TexMACS (Miltenyi Biotec) supplemented with 20 UI/mL of IL-2 (Miltenyi Biotec) for EGFP experiments or with 10 ng/mL of IL-7 and IL-15 (Miltenyi Biotec) for CAR experiments. T cells were activated with T cell TransAct (Miltenyi Biotec) during 48 h as recommended by the manufacturer.

### Ethics approval and consent to participate

All donors from the Haematology Unit of Hospital Reina Sofía, Córdoba (Spain) gave their written informed consent, and the study was performed according to the established guidelines in the approved project by the Regional Government Junta de Andalucía-Consejería de Salud (date of approval: 22/02/2019; signed by D^a^ Cristina Lucía Dávila Fajardo as secretary of the Research Ethics Committee), which comply with the requirements regarding quality and safety for donation, obtaining, storage, distribution, and preservation of human cells and tissues under the Spanish-specific regulation (RD 9/2014) and International Conference of Good Clinical Practice. All the experiments involving animals were performed according to a protocol approved by the Institutional Animal Care and Use Committee of the University of Granada (13/12/2016/181), in accordance with the European Convention for the Protection of Vertebrate Animals used for Experimental and Other Scientific Purposes (CETS no. 123) and the specific Spanish law (RD 53/2013). Subjects were randomly assigned to receive the different treatments, and sample-size estimation was calculated based on Mayer et al.^34^

### Plasmid constructs

To construct the SELWP vector, a self-inactivated (SIN) LV expressing EGFP and NanoLuc under the SFFV promoter, an EGFP-P2A-NanoLuc (NanoLuc sequence obtained from GenBank: JQ437370, nucleotides 100–616) flanked by AscI/SbfI restriction sites was designed and synthesized by GenScript (GenScript USA, NY, USA). The EGFP-P2A-Nluc was cloned into the SEWP LV^35^ by standard molecular biology techniques to obtain the SELWP.

We designed the EhCD19 to drive human CD19 (31–1,701 bp from NM_001178098-1 sequence) expression under the EF1α promoter based on VectorBuilder database’s recommendations.

We used self-inactivated, LVs-driven EGFP already available in our laboratory. SE,^19^ CEWP,^12^ and EFEWP (our laboratory) drive EGFP under the control of SFFV promoter, CMV, and the EF1α promoters, respectively. For *WAS*-promoter-based LVs, WE-LVs harbor 0.5 kb of the *WAS* proximal promoter,^19^ and the AWE-LVs include 0.5 kb of the *WAS* proximal promoter and 0.38 kb of the alternative promoter.^20^

For AWARI-LVs generation, the AW promoter was obtained from the AWE^20^ by ClaI/BamHI digestion and inserted into the ARI-0001 plasmid,^32^ replacing the EF1α promoter.

### Lentiviral vector production and titration

Lentiviral vectors were generated by co-transfection of HEK-293T cells with the plasmid of interest, the plasmid pCMVDR8.91, and the p-MD-G plasmid as previously described.^14^ LVs were concentrated by ultracentrifugation at 90,000 × *g* for 2 h at 4°C, resuspended in TexMACs, and stored at −80°C. LVs titters were determined by transducing Jurkat cells with different dilutions of viral supernatant. Percentage of positive cells was determined by flow cytometry, and transducing units per mL (TU/mL) were estimated according to the formula (10^5^ plated cells × % of positive cells)/mL of LV.

### T cell transduction

Activated primary T cells were transduced with the different LVs at a multiplicity of infection (MOI) of 10 through spinoculation (800 × *g* for 60 min at 32°C). Media were exchanged after 5 h of incubation. Four to six days after transduction, the percentage of transduced cells was determined by flow cytometry.

### Expression pattern analysis of LVs on T cells

For T cells transduced with EGFP-LVs, 10^5^ T cells were stimulated with TransAct (Miltenyi Biotec) after 10 days of the initial activation for transduction. Similarly, 10^4^ transduced CAR-T cells were stimulated with 10^4^ CD19^+^ Namalwa-GFP-Nluc cells in order to simulate the CAR/TCR signaling axis in U-bottom 96-well plates. EGFP or CAR expression was determined at different time points after stimulation. Cells were stained and fixed with 2% paraformaldehyde (PFA) prior to fluorescence-activated cell sorting (FACS) acquisition. Fold change of EGFP was indicated as the ratio of MeFI of positive population against the MeFI of non-transduced total population and those values related to 0 h. Fold change of CAR indicates the MeFI related to those values at 0 h.

### Flow cytometry

CAR expression was determined with a biotin-conjugated goat anti-murine Fab SP-longer Spacer immunoglobulin G (IgG) (1:40) (Jackson Immunoresearch, Philadelphia, PA, USA) and antigen-presenting cell (APC)-conjugated streptavidin (1:350) (Thermo Fisher Scientific, Waltham, MA, USA). Cells were washed with 2 mL of FACS buffer (PBS + 2% BSA + 2 mM EDTA) between each step. For human T cell phenotyping, the following monoclonal antibodies (mAbs) were used: CD45RA-phycoerythrin (PE)/fluorescein isothiocyanate (FITC) (HI-100, 1:400), CD62L-PE-Cy7 (DREG56, 1:400), CD3-PerCP-Cy5/APC-780 (OKT3, 1:400), PD1-Allophycocyanin (APC) (MIH4, 1:200), LAG-3-PE (3DS223H, 1:200), TIM-3-APC-Cy7 (F38-2E2, 1:200), and CD95-APC (Fas/ApoI, DX2, 1:100), all from eBioscience (Thermo Fisher Scientific). Tonic signaling was determined by intracellular staining with pCD3z-PE (Tyr142, 3ZBR4S, 1:100) and Fix & Perm Kit (Nordic MuBio, Susteren, the Netherlands). Samples were acquired on a FACSCantoII cytometer (BD Dickinson, NJ, USA). FlowJo software (TreeStar, BD Biosciences, USA) was used for data analysis.

### Cytokine secretion of CAR-T cells

To analyze cytokine production after T cells stimulation, 5 × 10^4^ CAR-T cells were co-cultured with Namalwa-GFP target cells at ratio 1:1 in TexMACs without any supplement. Supernatants were collected after 24 h and frozen at −80°C. TNF-α and IFN-ɣ were measured with anti-human TFN-α Ready-SET-Go! Kit of Affymetrix (eBioscience) and ELISA MAX Deluxe Set (BioLegend, San Diego, CA, USA), respectively, following manufacturer’s instructions.

### Cytotoxicity assay

Target cells (CD19^+^), such as Namalwa and Nalm-6 cells expressing GFP-Nluc and non-target cells (CD19^−^) HL60 cells (unlabeled), were co-cultured at a concentration of 5 × 10^3^ cells per well in duplicate in 96-well U-bottom plates and incubated with CAR-T cells at various effector-target (E:T) ratios (0.5:1, 1:1, and 2:1) in non-supplemented TexMACs during 16 h or 48 h, as indicated. Percentage of lysis was determined by flow cytometry related to basal lysis produced by non-transduced T cells. Specific lysis was determined as follows: 1 − (% CD19^+^/% HL-60 in CAR-T cells condition/% CD19^+^/% HL-60 in NTD condition) × 100.

### CD19^+^ MiaPaCa2 persistence assays

To determine specific lysis with the MiaPaCa2 cells, 1.5 × 10^4^ CD19^+^ and CD19^−^ pancreatic cells were seeded in wells of a 96-well plate with a 200 μL final volume of DMEM supplemented with 10% fetal bovine serum (FBS) and 1% P/S. At 24 h, the medium was aspirated and the CAR-T cells were co-cultured in a 5:1 ratio (CAR-T:target) in non-supplemented TexMACs medium. After 48 h, specific lysis and phenotypic markers CD45RA and CD62L were analyzed by FACS. Twenty microliters of the supernatant was transferred to the cytometry tube, and the remaining volume of the surviving T cells was transferred to a new well with already attached 1.5 × 10^4^ CD19^+^ and CD19^−^ pancreatic cells. All the remaining MiaPaCa2 cells were detached with Tryple 1x (Gibco) for 7 min at 37°C and transferred to the previous cytometry tube for analysis. Percentage of GFP-MiaPaCa2 cells CD19^+^ or CD19^−^ of the different conditions were considered to determine the specific lysis as follows: 1 − (% MiaPaCa2−CD19^+^/MiaPaCa2−CD19^−^ in CAR-T cells condition/% MiaPaCa2−CD19^+^/MiaPaCa2−CD19^−^ in NT condition) × 100.

### *In vivo* xenograft animal model and bioluminescence analysis

Ten- to twelve-week-old non-obese diabetic (NOD)/severe combined immunodeficiency (SCID)-IL-2Rnull (NSG) mice were inoculated intravenously with 3 × 10^5^ Namalwa-GFP-Nluc cells per mice, and 3 days later, mice were randomly infused with bulk CAR-T cells (5 × 10^6^ or 10 × 10^6^), non-transduced T cells (5 × 10^6^ or 10 × 10^6^) or PBS in the tail’s vein. In all the cases, ∼1–1.5 × 10^6^ of CAR^+^ cells were infused (low dose: ∼1.26 [ARI] and 1.12 × 10^6^ [AWARI] CAR-T cells and high dose: ∼1.47 [ARI] and −1.32 × 10^6^ [AWARI] CAR-T cells). Rechallenge was assessed by re-inoculating intravenously a new dose of Namalwa-GFP-Nluc cells. Our experiments are based on a modified randomized block design in which each block receives more than one treatment at different periods. Subjects were randomly assigned to receive the different treatments (PBS, T cells, or CAR-T cells). This will allow a comparison between the different treatment groups in pairs (each one compared with the control and each one compared with each other). For the estimation of the sample size in each experiment, we calculated the sample size necessary to obtain a p ≥ 0.05 to be of n ≥ 5 for controls and CAR-T comparison and an n ≥ 8 for AWARI and ARI comparisons based on Mayer et al.^34^

For bioluminescence analysis, furimazine (Nano-Glo, Promega Biotech, Madison, WI, USA) was diluted at 1/60 in PBS and injected intraperitoneally immediately prior to acquisition on an IVIS Spectrum analyzer (Caliper, PerkinElmer, Waltham, MA, USA). Images were acquired during 180 s, open field, and analyzed using the Living Image 3.2 (PerkinElmer) or AURA Imaging Software 3.2 (Spectral Instruments Imaging, Tucson, USA). Mice were sacrificed if experiencing a weight loss greater than 20% or at the indicated days. Samples of blood, bone marrow, liver, spleen, and brain were collected. Blood was extracted and diluted 1/5 in EDTA. Cells were obtained from liver and spleen by mechanical disruption and from bone marrow by the perfusion of both femurs and tibias. Brain’s cells were obtained after Percoll-gradient separation. Percentage of surviving Namalwa-GFP-Nluc and T cells (hCD3^+^) was determined by FACS in the singlet’s gate and then in “human cells” gate, which was previously established after acquiring an artificial mixture of Namalwa cells and human T cells used as control.

### GMP-like manufacturing of CAR-T cells on CliniMACs prodigy

Large-scale manufacturing of CAR-T cells on CliniMACs Prodigy was carried out under GMP-like conditions into Gene-Cell Therapy clean rooms of the Cell Therapy Unit of Hospital Universitario Reina Sofía (Córdoba, Spain). Two different aphereses from a healthy donor were thawed, and around 100 × 10^6^ T cells were inoculated into the CliniMACs prodigy bioreactor (Miltenyi Biotec). CD4 and CD8 cells were selected with CD8 and CD4 Reagent (Miltenyi Biotec), cultured with IL-7 and IL-15 (Miltenyi Biotec), and activated with αCD3 andαCD28 GMP T cell TransAct (Miltenyi Biotec). On day 2 of the process, these cells were transduced with AWARI-LVs (MOI = 5). Cells were cultured in TexMACs GMP medium containing GMP-grade IL-15 and IL-7 (Miltenyi Biotec) for 9 or 10 days. Final product was collected with 100 mL of NaCl 0.9% + 0.5% human serum albumin (HSA).

Cells were stained with CD3-APC, CD4-FITC, CD8-APC Vio770, CD14-PE Vio770, and CD45-Vioblue (Miltenyi Biotec). To assess the efficiency of transduction, CAR-T cells were stained with CD-19 Biotin and anti-Biotin PE (Miltenyi Biotec). Viability was tested by using 7-AAD (Miltenyi Biotec). Phenotype was determined with CD45RA-APC (HI100) and CCR7-BV421 (2-L1-A RUO) from BD Pharmingen. Cells were acquired on a MACsQuant cytometer (Miltenyi Biotec) and analyzed with MACsQuantify Analysis Software (Miltenyi Biotec).

### Data analysis

Statistical analyses were performed using GraphPad 9 software (GraphPad Software, La Jolla, CA, USA). Data were expressed as the mean ± SEM. Each performed statistical test was indicated in every figure caption. Survival curves were constructed using the Kaplan-Meier method.

## Supplemental information

Document S1. Figures S1–S5 and supplemental materials and methods

Document S2. Article plus supplemental information
