# Physiological lentiviral vectors for the generation of improved CAR-T cells

**DOI:** 10.1016/j.omto.2022.07.005

**Published:** 2022-07-21

**Authors:** María Tristán-Manzano, Noelia Maldonado-Pérez, Pedro Justicia-Lirio, Pilar Muñoz, Marina Cortijo-Gutiérrez, Kristina Pavlovic, Rosario Jiménez-Moreno, Sonia Nogueras, M. Dolores Carmona, Sabina Sánchez-Hernández, Araceli Aguilar-González, María Castella, Manel Juan, Concepción Marañón, Juan Antonio Marchal, Karim Benabdellah, Concha Herrera, Francisco Martin

## Main text

(Molecular Therapy: Oncolytics *25*, 335–349; June 2022)

Due to a supplier copyediting error, in the originally published version of this article, the GMP acronym in the abstract was defined as “guanosine monophosphate,” and in all the cases in this article the meaning should be “good manufacturing practice.”

In addition, the ScFv clone of ARI-0001 should be A3B1 instead of A1B6 as indicated in the first result.

These errors have been corrected online. Cell Press apologizes for any inconvenience caused.

