# Supplementary material for: Physiological lentiviral vectors for the generation of improved CAR-T cells
Source: Mol Ther Oncolytics. 2022 May 18;25:335–49. doi: 10.1016/j.omto.2022.05.003 (PMC9163403; doi:10.1016/j.omto.2022.05.003)
Supplement: Document S1. Figures S1–S5 and supplemental materials and methods [file mmc1.pdf]

OMTO, Volume *25*

Supplemental information

Physiological lentiviral vectors

for the generation of improved CAR-T cells

María Tristán-Manzano, Noelia Maldonado-Pérez, Pedro Justicia-Lirio, Pilar Muñoz, Marina Cortijo-Gutiérrez, Kristina Pavlovic, Rosario Jiménez-Moreno, Sonia Nogueras, M. Dolores Carmona, Sabina Sánchez-Hernández, Araceli Aguilar-González, María Castella, Manel Juan, Concepción Marañón, Juan Antonio Marchal, Karim Benabdellah, Concha Herrera, and Francisco Martin

Supplemental Figures and Materials

Title

Physiological (TCR-like) regulated lentiviral vectors for the generation of improved CAR-T cells

María Tristán-Manzano[1,2*#], Noelia Maldonado-Pérez[1*], Pedro Justicia-Lirio[1,2], Pilar Muñoz[1,3], Marina Cortijo-Gutiérrez[1], Kristina Pavlovic[1,3], Rosario Jiménez-Moreno[4], Sonia Nogueras[4], M. Dolores Carmona[4], Sabina Sánchez-Hernández[1], Araceli Aguilar-González[1,5], María Castella[6], Manel Juan[6], Concepción Marañón[1], Juan Antonio Marchal[7], Karim Benabdellah[1], Concha Herrera[4] and Francisco Martin[1,8#].

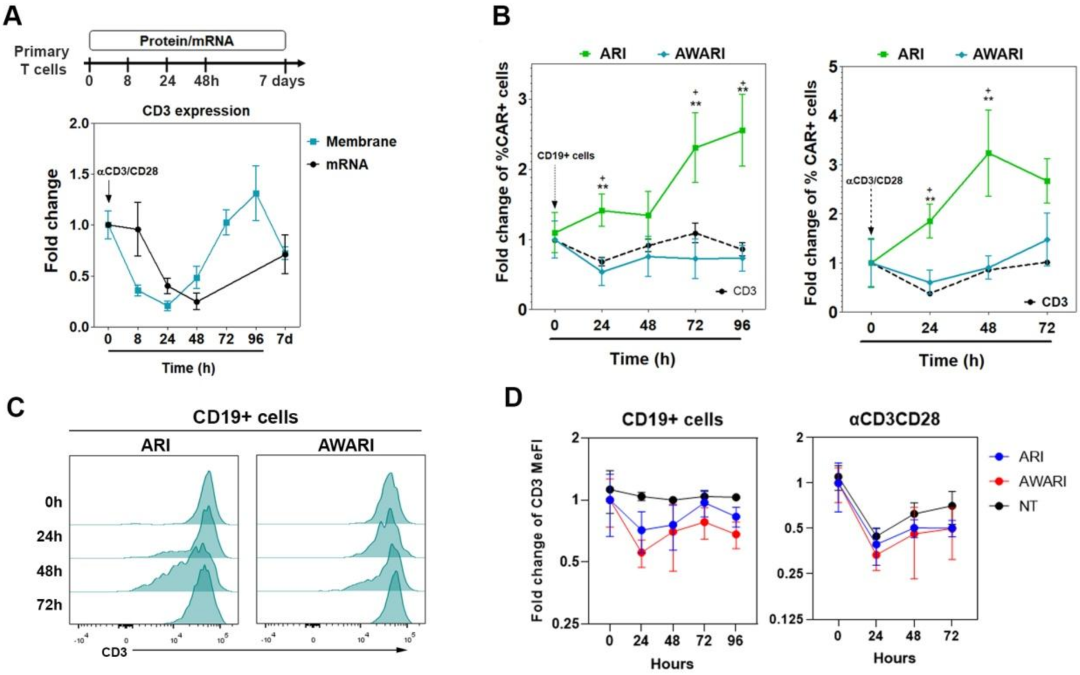

Supplementary Figure 1. A) CD3 kinetics after aCD3/CD28 stimulation at protein (membrane, n=10) and at mRNA level (n=5). **B**) Fold change of the percentage of CAR+ cells driven by EF1-α (green) and AW-CAR19-BBzz (blue) CAR-T cells stimulated with CD19+ cells (**left**) and αCD3/CD28 (**right**) compared to CD3 expressionpattern (black). Fold change indicates the percentage at different times related to those of 0h. ANOVA test, Bonferroni Post-test. *, $p<0.05$**. C)** Histograms of CD3 expression of ARI and AWARI CAR-T cells after the stimulation with CD19+ cells along the time. **D**) Fold change of CD3 MeFI of Non-transduced T cells (NT, black) and ARI (blue) and AWARI (red) CAR-T cells after the stimulation with CD19+ (left) or αCD3/CD28 (**right**).

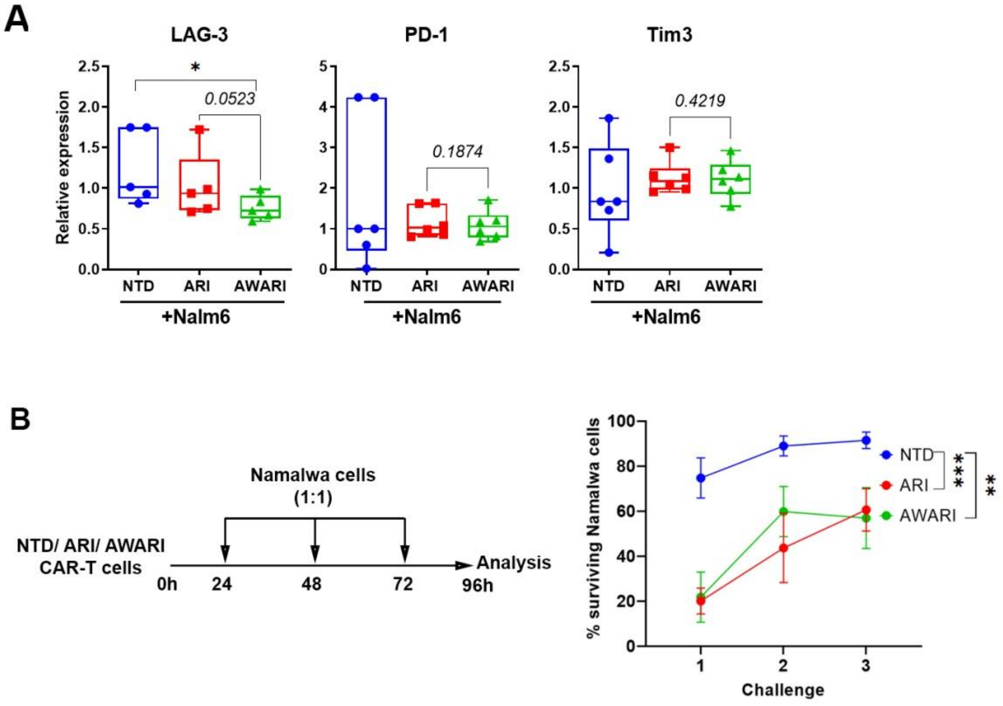

Supplementary Figure 2. A) Relative expression of LAG-3, PD-1 and Tim3 markers driven by ARI and AWARI CAR-T cells after being co-cultured with Nalm6 during 48h. Relative expression is referred to MeFI levels of every NT donor. One-tailed paired T-test (n≥5). **B**) Left: work-flow of the anti-lymphoma efficacy of NT, ARI and AWARI CAR-T cells after with three challenges with Namalwa cells at an initial ratio 1:1 target:CAR-T cells. Right: percentage of surviving Namalwa CD19+ tumor cells in the culture after one, two or three challenges over NT or CAR-T cells. 2-way ANOVA, Tukey’s multiple comparisons.

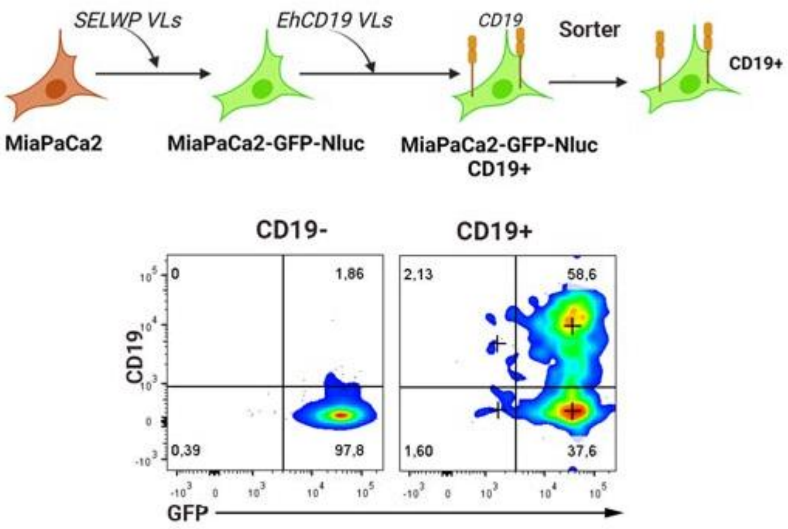

Supplementary Figure 3

Top: Diagram of CD19+GFP+Nluc+MiaPaCa2 model's generation. MiaPaCa2 cells were first transduced with SELWP to generate GFP+NLuc+ cells. Then, the bulk population was transduced with EhCD19 LV and GFP+CD19+ cells were sorted. Bottom: Dot-plots of CD19- and CD19+ GFP+Nluc MiaPaCa2 cells used in the performed assays. Note that CD19 was downregulated or silenced in the sorted population by the time of the experiments.

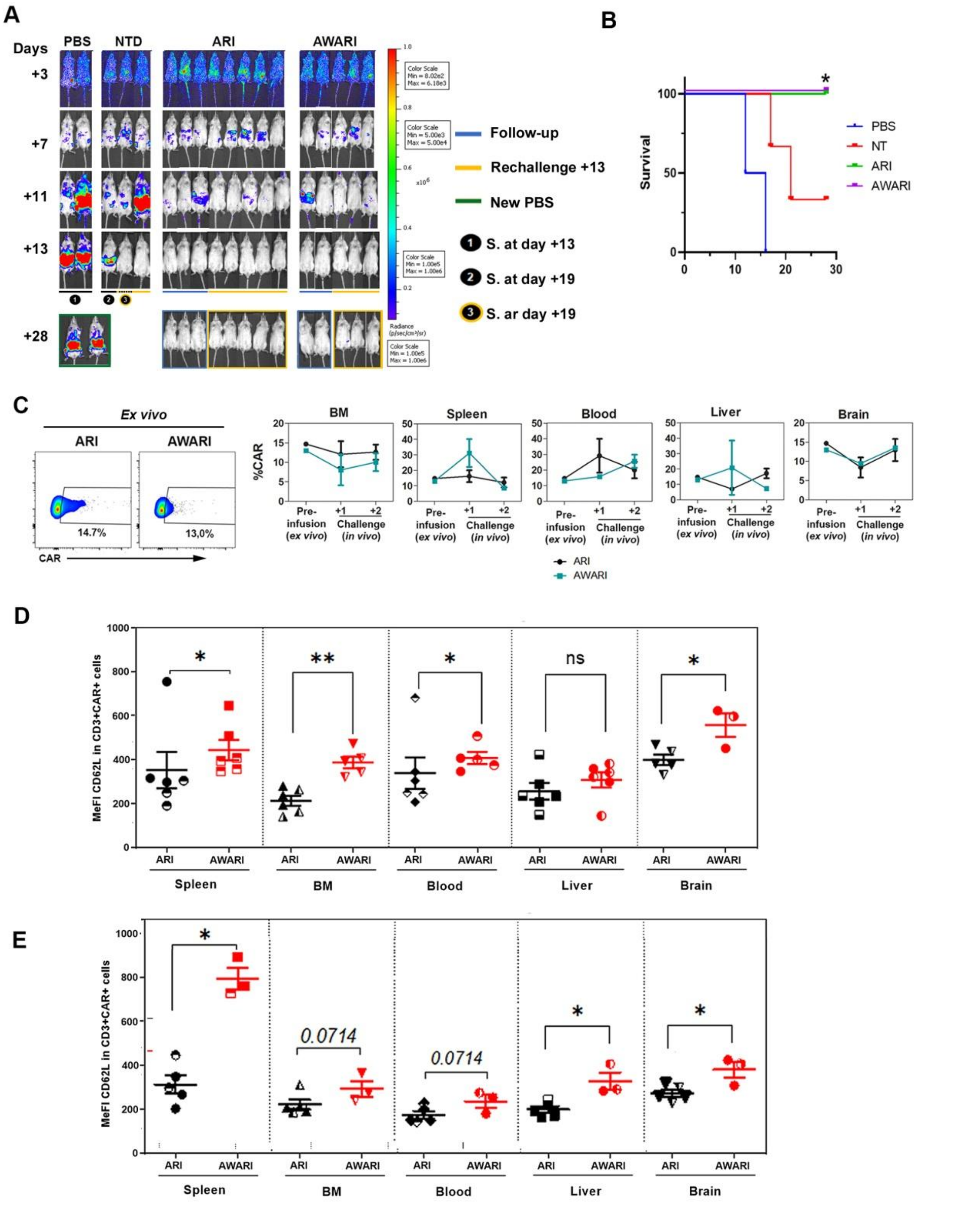

Supplementary Figure 4. A) BLI images of PBS, NTD, ARI and AWARI treated mice for up to 28 days. Rechallenged mice are highlighted in yellow. **B**) Survival of the different groups of mice. Mantel-Cox test, * indicates *p=0,0476.* **C) Left:** Dot-Plots of CAR expression of ARI and AWARI CAR-T cells *ex vivo*, prior to the infusion into de the mice. **Right:** Percentage of CAR+ cells analyzed in the hCD3+ population in the different organs from ARI and AWARI treated-mice after sacrificed one (+1) or two (+2) challenges with tumor cells compared to *ex-vivo* percentage before the infusion. No significance, 2-way ANOVA, Tukey's multiple comparisons. **D**, **E**) MeFi of CD62L in hCD3+CAR+ cells infiltrated in the different organs of mice with one (**D**) or two (**E**) Namalwa challanges. Half-full dots indicate mice from the 5x10^{6}dose experiment. Only when CAR+ population was >1%, data was analyzed and included here. One-tailed nonparametric T test.

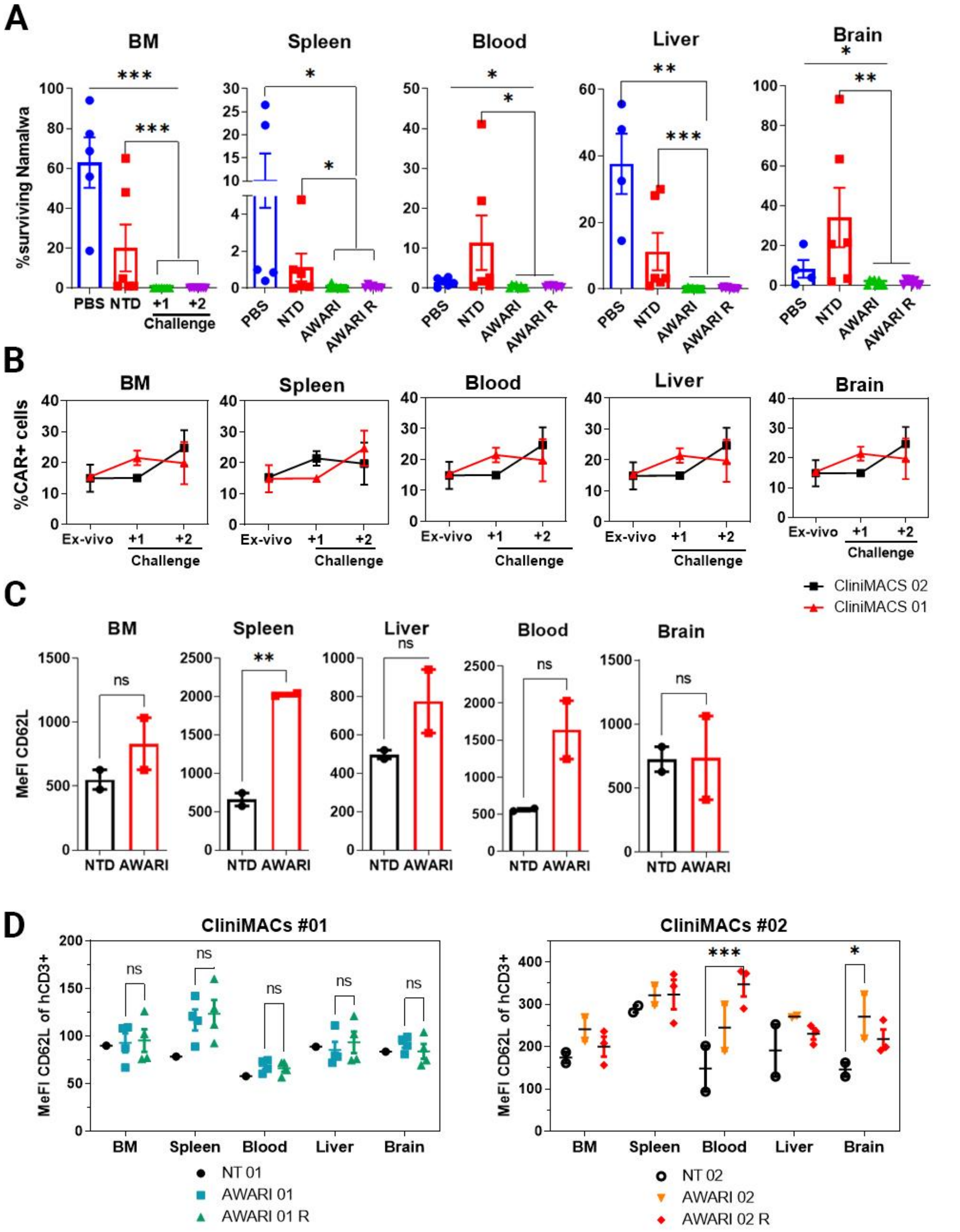

Supplementary Figure 5. A) Percentage of surviving Namalwa in both #01 and #02 CliniMACS experiments with one or two Namalwa inoculations analyzed by FACS at final points. One-tailed Mann-Whitney T- test. **B**) Percentage of CAR+ cells analyzed in the hCD3+ present in the different organs from mice inoculated with one or two challenges with tumor cells compared to *ex-vivo* percentage of AWARI CliniMACs #01 and #02 before the infusion. **C**) CD62L MeFI of hCD3+ from different analyzed organs of NT and AWARI CliniMACS #01 at day 13 post-inoculation. Two-tailed T-test. **D**) CD62L MeFI of hCD3+ from different analyzed organs of NT and AWARI mice with one or two (rechallenged, R) tumor inoculations of #01 (left) and #02 (right). 2-way ANOVA, Tukey's multiple comparisons.

Material and Methods

RNA extraction and quantitative PCR

Total RNA from T cells was extracted using Trizol (Ambion) and RNA samples were converted into cDNA using the high-capacity cDNA reverse transcription kit (ThermoFisher), complemented with RNase inhibitor (ThermoFisher). Q-PCR was performed in a Stratagene MX3005P system, using the Kappa SYBR FAST pPCR Master Mix (Kappa Biosystems) and primers against CD3: CD3-Fw 5´-AAGATGAAGTGGAAGGCG-3´ and CD3-Rv 5´-CTCAGGAACAAGGCAGTG-3´. GAPDH as housekeeping gene: GAPDH-Fw 5´-ATGGGGAAGGTGAAGGTCG-3´ and GAPDH-Rv 5´-GGGGTCATTGATGGCAACAATA-3´. Relative changes in gene expression were analysed using the 2^ΔΔCt method
